# The role of sensory experience in the maturation of prefrontal cortical circuits

**DOI:** 10.3389/fnins.2026.1808558

**Published:** 2026-04-21

**Authors:** Amanda Anqueira-González, Sarah Canetta

**Affiliations:** 1 Division of Developmental Neuroscience, Department of Psychiatry, Columbia University Irving Medical Center and the New York State Psychiatric Institute, New York, NY, United States; 2 Doctoral Program in Neurobiology and Behavior, Columbia University, New York, NY, United States; 3 Columbia University Institute for Developmental Science, Columbia University Irving Medical Center and the New York State Psychiatric Institute, New York, NY, United States

**Keywords:** environmental enrichment, flexible behavior, goal-directed, sensory integration, sensory restriction, prefrontal cortex, development

## Abstract

Sensory input during early life is crucial for brain circuitry to be appropriately wired and refined. Foundational studies in the past century established that early sensory input was required for the appropriate development of primary sensory areas. Further investigation in the beginning of the 21st century extended this idea by suggesting that early sensory inputs may also impact remodeling of associative cortical regions. While many of the early studies promoting this idea were based on correlational observations, more causal studies followed soon after. It quickly became clear that sensory experience is a driver for shaping associative regions, including those that do not necessarily receive direct sensory input, such as the prefrontal cortex (PFC). The PFC is a region critical for sensory integration as well as for goal-directed, flexible behavior across species. Importantly, the PFC is a late developing structure, where the integration of diverse types of information, such as sensory information, during early life can elicit alterations in the underlying developing neural circuitry. These sensory inputs can interact with genetically-encoded biological programs to shape the maturation of PFC circuitry. In this review, we will highlight the studies supporting this model and delve further into how sensory experience during early life can impact different biological mechanisms to shape developing PFC circuitry.

## 1 Introduction

Sensory input during early life is crucial for brain circuitry to be appropriately wired and refined. Foundational studies in the past century established that early sensory input was required for the appropriate development of primary sensory areas (Knudsen and Brainard, 1991; Van Der Loos and Woolsey, 1973; Wiesel and Hubel, 1963). Further investigation in the beginning of the 21st century extended this idea by suggesting that early sensory inputs may also impact remodeling of associative cortical regions. While many of the early studies promoting this idea were based on correlational observations, more causal studies followed soon after. It quickly became clear that sensory experience is a driver for shaping associative regions (Bavelier and Neville, 2002; Bengoetxea et al., 2012), including those that do not necessarily receive direct sensory input, such as the prefrontal cortex (PFC).

The PFC is a region critical for sensory integration as well as for goal-directed, flexible behavior across species (Cao et al., 2024; Friedman and Robbins, 2022; Goodpaster et al., 2026; Menon and D’Esposito, 2022; Murray and Constantinidis, 2024). Importantly, the PFC is a late developing structure, where the integration of diverse types of information, such as sensory information, during early life can elicit alterations in the underlying developing neural circuitry (Kolb et al., 2012; Kolk and Rakic, 2022). These sensory inputs can interact with genetically-encoded biological programs to shape the maturation of PFC circuitry. In this review, we will highlight the studies supporting this model and delve further into how sensory experience during early life can impact different biological mechanisms to shape developing PFC circuitry.

## 2 Overview of prefrontal cortical development and maturation

Compared to sensory and motor areas, prefrontal circuitry has been proposed to have a more protracted period of physiological maturation, potentially elongating the susceptibility of the PFC to sensory input. Below, we summarize general stages of cellular neurodevelopment relevant to the formation and maturation of the PFC. Initially, a series of genetic mechanisms lay the foundational blueprint for the structural organization of the PFC which is subsequently refined based on interactions with experience. Although the general pattern of cellular neurodevelopment is quite similar across rodents and humans, there are some distinctions, especially regarding which processes occur pre- versus postnatally, which may lead to potential differences in the way that postnatal sensory experience can impact brain development in these different organisms. Defining prenatal versus postnatal developmental stages across species is inherently challenging, as equivalent neurodevelopmental events often span different temporal windows relative to birth complicating direct cross-species comparisons. Therefore, for the purposes of this review, when discussing findings from model organisms, we will refer to specific ages while also referencing their approximate stage of development.

Neural development supporting PFC formation unfolds along a continuous trajectory marked by overlapping molecular and cellular events. Early in this trajectory, the neural plate folds to form the neural tube, a process called neurulation, establishing the architecture of the basic nervous system. Shortly after, molecular patterning cues specify anterior-posterior identity, including the emergence of frontal cortical territories through forebrain patterning, during which the future PFC is distinguished from the surrounding tissue (Kolb et al., 2012; Kolk and Rakic, 2022; Uytun, 2018). As development progresses, radial glial cells within the cortical ventricular zones proliferate and give rise to neurons destined for the PFC. These neurons undergo radial migration in an inside-out manner, assembling a layered cortical structure as excitatory glutamatergic projection neurons populate deeper layers before later-born neurons settle more superficially. In parallel, inhibitory GABAergic interneurons integrate into frontal cortical circuits arriving via tangential migration in rodents (Kelsom and Lu, 2013). In humans and non-human primates, interneuron origin appears to be more heterogeneous, involving both tangential and radial migration, although precise developmental trajectories are still incompletely understood (Lim et al., 2018; Petanjek et al., 2009).

Following neural positioning, neurons elaborate axons and dendrites and begin forming synaptic contacts, initiating synaptogenesis and the emergence of frontal cortical circuitry (Chini and Hanganu-Opatz, 2021; Pressler and Auvin, 2013; Zeiss, 2021). Here, a transient layer beneath the cortical plate, named the subplate, facilitates ingrowth of afferents and efferents in the PFC (Hadders-Algra, 2018; Luhmann et al., 2018). Importantly, the subplate has been shown to be thicker in the PFC compared to other cortical regions, and its ablation leads to impairments in inhibitory maturation, suggesting a key role in PFC refinement (Kolk and Rakic, 2022; Lee and Rajakumar, 2022). During this stage, neural networks increasingly incorporate experience-driven activity, which promotes dendritic arborization and spine formation in PFC neurons (Chomiak and Hu, 2017; Kroon et al., 2019; Oga et al., 2017; Song and Moyer, 2018). In contrast to the relatively early stabilization of excitatory connectivity, inhibitory network maturation, including GABAergic transmission strength and interneuron functional integration, continues through later stages, reflecting a more protracted developmental trajectory (De Marco García et al., 2011; Gonzalez-Burgos et al., 2015; Kim and Paredes, 2021; Le Magueresse and Monyer, 2013; Lim et al., 2018; Okaty et al., 2009). As circuit complexity increases, synapse and spine formation transiently exceeds adult levels, producing a period of heightened synaptic density that can be approximately 40% greater than what is observed in maturity (Thompson, 2024). This surplus is subsequently refined through synaptic pruning, a process that selectively eliminates weaker or redundant connections, and neuronal apoptosis, aimed at reducing cells that fail to form strong connections within the circuit (Fleming and McDermott, 2024; Sellinger et al., 2021). Together, these processes conduct tissue removal with the purpose of enhancing the efficiency and specificity of information transfer within local and long-range circuits (Levman, 2025). In parallel, myelination of PFC axons progresses, providing metabolic support and increasing the speed and reliability of neuronal communication (Fletcher et al., 2021; Hettwer et al., 2024; Miller et al., 2012). Myelination, initiated early and extending across development, has emerged as a key contributor to cognitive maturation (Khelfaoui et al., 2024; Nickel and Gu, 2018; Zhang et al., 2025), with recent evidence indicating that disruptions to myelin during developmental windows impairs the maturation and function of parvalbumin-expressing interneurons in the PFC (Hijazi et al., 2025). Together, these coordinated and overlapping processes culminate in the stabilization of synaptic connectivity, inhibitory-excitatory balance, and neuromodulatory systems that support mature executive function.

While many of the afore-described neurodevelopmental events are under the control of genetic programming, experience, especially sensory experience that is heightened in the postnatal period, also plays an important role in the unfolding of many of these processes. Notably, the effects of experience can differ depending on the developmental window in which the experiences occur, a concept that is referred to as sensitive periods. Sensitive periods in which experience can exert lasting effects on cortical functioning were classically described in the visual system through the pioneering work of Hubel and Wiesel (1970). They identified a sensitive period in which early visual experience is required for proper visual cortical connectivity (Hubel and Wiesel, 1970; Wiesel and Hubel, 1963). Mechanistically, the effects of visual experience on visual cortical connectivity arise due to changes in neuronal activity (Hensch, 2005). This concept of sensitive periods for activity-dependent wiring of cortical circuitry has been expanded to the PFC. Indeed, recent studies in mice demonstrate that the timing of activity manipulations is critical, such that altering PFC activity during specific developmental windows, via long range projections to the PFC from postnatal day (PN) 20 to PN35 (early adolescence) (Benoit et al., 2022; Petersen et al., 2024) or specific cell types within the PFC from PN14-PN32 (juvenile and early adolescence) (Bitzenhofer et al., 2021; Canetta et al., 2022; Sahyoun et al., 2024), impacts PFC maturation, with long-lasting effects on adult function and PFC-dependent cognitive behavior. Although the PFC does not receive direct sensory inputs, sensory experiences can still drive changes in prefrontal activity. In this review, we summarize the evidence that changes in sensory experience can impact PFC maturation, including whether there are heightened sensitive periods for these effects.

## 3 External sensory-driven factors shaping PFC circuitry

In the adult, the PFC is essential for integrating various types of sensory as well as emotional and cognitive information. Although the PFC does not receive direct sensory inputs, it does receive projections from several different sensory cortical areas including somatosensory cortices, parietal visual areas, as well as auditory and temporal cortices (Bedwell et al., 2014; Cappe et al., 2012; Murray and Constantinidis, 2024; Romanski, 2012). In addition, it receives inputs from subcortical structures including the amygdala, hippocampus and thalamus that are believed to convey emotional as well as cognitive information. While the precise timing for the earliest arrival of projections from sensory association areas to the prefrontal cortex is not certain, it is clear that these inputs are present in the PFC by the time of birth, in both humans and rodents (Chen et al., 2025; Hoerder-Suabedissen et al., 2023; Taymourtash et al., 2023). Therefore, it is not surprising that changes in incoming sensory or emotional information from the postnatal environment could influence the development of the PFC. Below, we review studies that have examined how different types of early sensory or emotional experience influence PFC development. In each section we start with evidence from human studies when available and then delve into the richer and more detailed literature that is available from studies in model organisms.

### 3.1 Sensory restriction and enrichment

To address whether the sensory environment influences prefrontal cortical development, researchers have examined the impact of extreme versions of the sensory environment, including sensory restriction and environmental enrichment. One example is the devastating case of Genie, a girl confined to a severely restricted, windowless environment from 20 months of age until her discovery at 13 years old. Despite subsequent intensive intervention, Genie never acquired normal language abilities and showed persistent deficits in executive function, social interaction and emotional regulation, which are functions strongly associated with PFC circuitry, suggesting that sensory inputs are required for normal prefrontal development (Fromkin et al., 1974). However, this case represents an extreme form of sensory restriction and is heavily confounded by chronic stress, physical abuse and malnutrition, making it difficult to isolate the specific contribution of reduced sensory input to altered PFC development. As an alternative, researchers have examined more mild examples of sensory restriction, such as individuals born without sight or hearing. On this front, studies have demonstrated reorganization in functional connectivity among different brain regions, including the PFC, following congenital visual or hearing impairments, consistent with the idea that developmental sensory experience shapes PFC connectivity (Hasson et al., 2016; Li J. et al., 2025; Tian et al., 2024; Xia et al., 2017; Yin et al., 2024).

The role of sensory experience in prefrontal development is more easily studied in model organisms. For example, researchers have found that sensory restriction, elicited by whisker trimming or dark rearing from birth in rodents, leads to increases in multiple subtypes of interneurons in a layer-specific manner in the prefrontal cortex (Ueno et al., 2015). Additionally, sensory restriction elicited by whisker trimming from birth to postnatal day PN35, results in impairments in prefrontal cortical myelination and oligodendrocyte complexity at PN60; this effect is not seen following whisker trimming from PN35-PN50 (He et al., 2024). The studies outlined above suggest that sensory restriction leads to alterations in developing PFC circuitry, consistent with similar effects that have been observed for sensory restriction to impact the development of sensory cortices (Barrera et al., 2013; Chen S. et al., 2024; Ueno et al., 2015).

In contrast to sensory restriction, environmental enrichment immerses animals in an environment enriched for sensory experiences including novel objects, complex textures, enhanced sensory stimulation, and opportunities for exploration and social interaction (Domínguez-Oliva et al., 2025; Figuracion and Lewis, 2021). Evidence from human as well as animal studies shows that environmental enrichment during development has profound impacts on neural maturation (Han et al., 2022; Tooley et al., 2021) and PFC-dependent cognitive behavior (Rosario et al., 2026). For instance, human studies show that exposure to enriching activities during early life results in alterations to cortical thickness and increased functional connectivity within subcortical areas (Brito and Noble, 2014; Lawson et al., 2013; Morris et al., 2021). This finding is consistent with work in rodents demonstrating that mice raised in an enriched environment, in particular from PN21-PN70 (juvenile to early adulthood), exhibit altered sensory-cognitive network connectivity and improvements to multisensory integration (You et al., 2025). Similarly, exposure to environmental enrichment within PN21-PN70 has been reported to result in increases in developing PFC volume and surface area, increases in proteins associated with synaptic plasticity, BDNF and PSD-95, improvements in interneuron myelination, and PFC-dependent cognition, with structural and functional effects typically observed in young adult animals (do Prado et al., 2016; González-Pardo et al., 2019; Kolb et al., 2012; Maas et al., 2020; Murack et al., 2023; Sadegzadeh et al., 2020; Tomlinson et al., 2018; Table 1). These findings suggest that the window PN21-PN70, encompassing “juvenile” and “adolescence,” may reflect an important period of development in which environmental enrichment can exert effects on PFC maturation. It is important to note that environmental enrichment during development has most often been investigated as a strategy to lessen the effects of prior disease or stress paradigms, underscoring its potential relevance as a therapeutic strategy (Bondi et al., 2014; do Prado et al., 2016; McCreary and Metz, 2016; O’Connor et al., 2024; Smith et al., 2018; Zhang et al., 2016; Zhou et al., 2025). An example of this is a study that showed that environmental enrichment from PN21 to PN56 (juvenile and adolescence) can prevent early life stress-induced increases in PFC pro-inflammatory cytokines and cognitive dysfunction (do Prado et al., 2016). Environmental enrichment can continue to influence prefrontal functioning in adulthood. Indeed, environmental enrichment in adulthood has been shown to modulate multiple mechanisms relevant to PFC function, including enhanced dendritic arborization and spine density, altered neuromodulatory signaling, epigenetic regulation of plasticity-related genes, and corresponding changes in PFC-dependent cognitive behavior (Ashokan et al., 2018; Darna et al., 2015; de Sousa Fernandes et al., 2024; Del Arco et al., 2007; Delarco et al., 2007; Ekstrand et al., 2008; Gelfo, 2019; Gomez et al., 2015; Segovia et al., 2009; Slaker et al., 2016). However, it remains unclear whether these same mechanisms are engaged during PFC development. Importantly, despite our understanding of one possible window, discrete sensitive periods during which different types of environmental enrichment exert maximal influence on PFC maturation are not clearly defined. Moreover, as previously mentioned, much of the literature examining changes induced by environmental enrichment in the developing PFC has been conducted within the context of stress exposure or disease models, leaving comparatively little work aimed at isolating the core biological processes through which environmental enrichment influences typical PFC development.

**TABLE 1:** Sensory experience-driven alterations to PFC development.

Model	Treatment/exposure	Window of developmental treatment/exposure	Approximate developmental stage	Findings on PFC development	References
Rodent (mouse)	Whisker trimming/dark rearing	PN0-PN28	Birth - juvenile	Increased interneuron subtype density (parvalbumin, calbindin and calretinin) in a layer specific-manner	Ueno et al., 2015
Rodent (mouse)	Whisker trimming	PN0-PN35	Birth - juvenile	Impaired PFC myelination and reduced differentiated oligodendrocytes	He et al., 2024
Human	Enriched environment (neighborhoods)	9–11 years old	Juvenile	Improved episodic memory, PFC task-based functional activity, increased PFC volume	Rosario et al., 2026
Human	Enriching early life activities (sport, art, music, volunteering, language learning, vacations)	Prior to the age of 13 years	Childhood - early adolescence	Increased efficiency in functional connectivity between orbitofrontal cortex, somatosensory regions and limbic networks	Morris et al., 2021
Human	Enriched socioeconomic status (parental education and family income)	4–13 years old	Childhood - early adolescence	Increased frontal gyrus thickness	Lawson et al., 2013
Rodent (rat)	Enriched environment (larger cages, socially stimulating environment, increased number of objects for play)	PN21-PN60	Juvenile - late adolescence	Increased PFC volume	González-Pardo et al., 2019
Rodent (rat)	Enriched environment	PN21-PN54	Juvenile - late adolescence	Prevention of stress induced PFC-dependent cognitive dysfunction and increase inflammatory cytokines	do Prado et al., 2016
Rodent (rat)	Enriched environment (increase in items to motivate exploration)	PN21-PN60	Juvenile - late adolescence	Increased PFC BNDF levels	Sadegzadeh et al., 2020
Rodent (mouse)	Enriched environment	PN21-PN42	Juvenile - early adolescence	Increased PFC BDNF and PSD-95 expression levels	Murack et al., 2023
Rodent (rat)	Enriched environment (increase in items to motivate play and exploration)	PN21-PN60	Juvenile - late adolescence	Promotion of PFC parvalbumin interneuron myelination and cognitive flexibility in schizophrenia model	Maas et al., 2020
Rodent (mouse)	Enriched environment (increased wheels for exercise)	PN28-PN63	Juvenile - late adolescence	Increase in NG2+ glial proliferation and elevated levels of glial lactate transporter, monocarboxylate transporter 1	Tomlinson et al., 2018

### 3.2 Stress and trauma exposure

Another type of sensory experience that is known to influence prefrontal cortical maturation is stress. Stressful experiences are highly heterogeneous, and during development their timing, severity, duration and physiological recovery can differentially shape neural maturation contributing to substantial variability in outcomes across individuals (Delarco et al., 2007; Ekstrand et al., 2008; Sheridan and McLaughlin, 2014; Birnie and Baram, 2025). Pioneering work on early life stress focused primarily on stress physiology, specifically the dysregulation of the hypothalamic-pituitary-adrenal (HPA) axis, adult behavioral outcomes resulting from stress exposure in development and mechanistic hypotheses centered around neuron and synapse dynamics. Over the past decade, both human and animal studies have been pivotal in contributing to our understanding of how the developing PFC is impacted by stress. In humans, exposure to stressors including childhood maltreatment, institutional rearing and socioeconomic deprivation across various developmental stages has been associated with altered PFC structure and function, including reductions in medial and dorsolateral PFC volume and thickness, inhibitory interneuron structure, disrupted functional connectivity, and altered engagement of the PFC during emotional regulation and cognitive control tasks (Jenness et al., 2021; Mackiewicz Seghete et al., 2017; McLaughlin et al., 2019; Park et al., 2018; Peverill et al., 2019; Silvers et al., 2016; Théberge et al., 2024; Zhong et al., 2020). Converging mechanistic insights from animal models indicate that, in the developing PFC, early life stress impacts functional connectivity in the PFC via alterations in inhibitory neuron functionality, including differential gene expression, reductions in GAD67 positive interneuron number and inhibitory synapses, reduced parvalbumin activity and levels, and reductions interneuron-associated perineuronal nets (Chen et al., 2025; Girgenti et al., 2019; Goodwill et al., 2018; Guadagno et al., 2021; Holland et al., 2014; Ma et al., 2025; Menezes et al., 2024; Ohta et al., 2020; Poleksic et al., 2021; Rahimian et al., 2024; Song et al., 2025). Alterations to prefrontal glia cell functionality are also found, including impairments in oligodendrocyte differentiation, decreased myelination, decreased astroglia number, increased microglia number, altered morphology and microglia-induced neuroinflammation in the PFC (Banqueri et al., 2019; Duan et al., 2025; Milbocker et al., 2021; Murasawa et al., 2023; Orso et al., 2023; Peña et al., 2019; Suwaluk and Chutabhakdikul, 2022; Tanti et al., 2018; Teissier et al., 2020; Yang et al., 2017). Chronic stress during PN28-PN42 in rodents (early adolescence), also delays PFC maturation by reducing dendritic complexity and spine density of pyramidal neurons, disrupting inhibitory parvalbumin interneurons and perineuronal net development, altering excitatory/inhibitory balance and changing gene expression, resulting in persistent deficits in adult PFC-dependent cognition (de Araújo Costa Folha et al., 2017; Ganzel et al., 2013; Page and Coutellier, 2018; Perica and Luna, 2023; Tyborowska et al., 2018). An important limitation of the existing literature is that although early life stress is experimentally manipulated, downstream outcomes are typically assessed in adulthood, obscuring when stress impacts on brain development initially manifest. Nevertheless, studies have begun to examine PFC maturation during or shortly following stress exposure (Donati et al., 2026; Menezes et al., 2024; Teissier et al., 2020), providing insight into changes in brain development that may emerge as the stress occurs. Moreover, how the sensory components of early life stress engage primary sensory circuits to drive downstream alterations in PFC development is still poorly understood.

### 3.3 Social experience

Social experience can be conceptualized as a specialized form of multisensory experience. In social experience, relevant auditory, visual, somatosensory and chemosensory cues are integrated and assigned value through higher order cortical circuits, including the PFC (de la Zerda et al., 2022). Moreover, PFC circuitry has been shown to be involved in aspects of complex social behavior including social recognition, aggression, empathy, and social hierarchy (Li H. et al., 2025; Mohapatra and Wagner, 2023; Yizhar and Levy, 2021). Considering the PFC’s role in guiding social behavior in adulthood, it is likely that social experience shapes PFC circuitry during development. Evidence from human studies suggest social experience during adolescence could be important in shaping the PFC, promoting appropriate development of socio-cognitive behaviors in adulthood (Larsen and Luna, 2018; Orben et al., 2020). Indeed, neuroimaging studies in human adolescents indicate that social experience engages and sculpts the developing PFC (Achterberg et al., 2022; Burnett et al., 2009, 2011; Grossmann, 2025; Qu et al., 2018; Schriber and Guyer, 2016; Stepanous et al., 2023; Welborn et al., 2016), with enriched and supportive social environments associated with increases in cortical surface area and thickness (Becht et al., 2021), whereas adverse social experience are linked to delayed or attenuated cortical thinning (Stepanous et al., 2023). Consistent with documented neurobiological alterations, human studies have also reported lasting changes in PFC-dependent behaviors following deprivation of early life social experiences (Wade et al., 2022; Will et al., 2016). However, adolescent social experience is complex and it is difficult to isolate the impact of specific aspects of the social environment on PFC maturation in humans, as well as how those effects may vary depending on their developmental timing.

As a result, much of what is currently understood about how social experience during adolescence shapes PFC circuitry has emerged from controlled animal studies, allowing precise manipulation of social environment across defined developmental windows (Yizhar and Levy, 2021). A substantial body of rodent work has used social isolation or deprivation to demonstrate that social experience is required for normal maturation of the PFC (Table 2). Disruption of social experience across adolescent and juvenile periods produces convergent but age-specific alterations in PFC circuitry. Social experience occurring from PN21-PN35/42 (approximately juvenile and early adolescence) was shown to be important for inhibitory circuit maturation, as social isolation during this time alters parvalbumin interneuron properties, increases inhibitory input onto pyramidal neurons, and heightens fast-spiking interneuron excitability (Okamura et al., 2023; Yamamuro et al., 2018, 2020). Extending isolation slightly later from PN28-PN63 (juvenile to early adulthood) further compromises parvalbumin interneuron number and activity, as well as cognitive flexibility (Bijlsma et al., 2022; Li et al., 2022). Across overlapping windows, social isolation consistently disturbs dopaminergic regulation in the PFC. Isolation from PN21-PN42 (juvenile and early adolescence) or shorter windows from PN30-PN35 (early adolescence) reduces dopaminergic modulation, decreases PFC D2 receptor expression and alters molecular pathways linked to drug vulnerability (Burke et al., 2017; Cuesta et al., 2020; Fitzgerald et al., 2013; Funes et al., 2025). Longer deprivation from PN28-56 (juvenile to late adolescence) produces similar reductions linking developmental social experience to enduring changes in dopamine signaling (Zhang et al., 2021). Structural plasticity outcomes, including dendritic and myelin-related changes, appear to be tied to prolonged or later deprivation. Isolation spanning PN28-56 diminishes dendritic complexity (Larsen and Luna, 2018), whereas deprivation extending from PN21-PN65 (juvenile to late adolescence) leads to reduced myelination and broader circuit architecture disruptions (Makinodan et al., 2012; Pais et al., 2019). Consistent with this, isolation in mice from PN31-PN56 (juvenile to late adolescence) produces excessive dendritic spine density, heightened network excitability and dysregulation of cytoskeletal pathways governing spine structure, accompanied by deficits in flexible, goal directed and rewards-related behaviors (Li et al., 2024). Together, these findings suggest that social deprivation in rodents during windows approximating juvenile to adolescent development alters multiple, temporally sensitive processes in PFC maturation. Reintroducing enriched or typical social environments leads to partial recovery of some functional disruptions (Hinton et al., 2019; Makinodan et al., 2017). These advances have provided great insight into how the PFC is shaped by early life social experience. However, further research is needed to elucidate how positive or enriched social environments (Popa et al., 2020) affect the developing PFC as well as how different dimensions of social experience drive particular cell-type or circuit changes impacting prefrontal maturation.

**TABLE 2:** Social experience-driven alterations to PFC development.

Model	Treatment/exposure	Window of developmental treatment/exposure	Approximate developmental stage	Findings on PFC development	References
Human	Social and motivational context	13–15 years old	Early adolescence	Increased ventrolateral PFC activation during cognitive control task	Qu et al., 2018
Human	Social scenario thinking	10–18 years old	Adolescence	Increased activation of lateral medial PFC during social thinking	Burnett et al., 2009
Human	Social influence task	16–18 years old	Adolescence	Social influence-evoked activity of PFC (medial PFC, ventromedial PFC, right ventrolateral PFC)	Welborn et al., 2016
Human	Social media use	11–24 years old	Adolescence - young adulthood	Higher social media usage correlates with higher baseline lateral and medial PFC thickness	Achterberg et al., 2022
Human	Social experiences (peer problems, family socioeconomic stress)	14–19 years old	Adolescence	Social experiences (peer problems) promote changes to ventromedialPFC gray matter volume and thinning	Stepanous et al., 2023
Human	Social experience (friendship quality)	8–25 years old	Adolescence - young adulthood	Stronger increases in friendship quality predicted by high levels of PFC medial PFC surface area, cortical thickness and faster cortical thinning	Becht et al., 2021
Human	Peer rejection and social exclusion	6–12 years old	Childhood	Higher anterior PFC activity upon social exclusion in adolescents with a history of childhood peer rejection	Will et al., 2016
Rodent (mouse)	Social isolation	PN21-PN35	Juvenile - early adolescence	PFC PH pyramidal cell action potential property deterioration, reductions in excitatory synaptic inputs and AMPA/NMDA ratio	Yamamuro et al., 2018
Rodent (mouse)	Social isolation	PN21-PN35	Juvenile - early adolescence	Increase in excitatory inputs to PFC parvalbumin interneurons and firing reactivity of PFC parvalbumin.	Okamura et al., 2023
Rodent (mouse)	Social isolation	PN21-PN35	Juvenile - early adolescence	Increased inhibitory inputs to PFC PH pyramidal cell, deterioration of action potential properties and increased excitability in fast spiking interneurons	Yamamuro et al., 2020
Rodent (mouse)	Social isolation	PN28-PN63	Juvenile - late adolescence	Increase in aggressive behavior, chemogenetic activation PFC parvalbumin interneurons alleviates isolation-induced aggressive behavior	Li et al., 2022
Rodent (rat)	Social play deprivation	PN21-PN42	Juvenile - early adolescence	Reduced inhibitory synaptic currents in the PFC, more reversals in a probabilistic reversal learning (PRL) task	Bijlsma et al., 2022
Rodent (rat)	Social isolation	PN30-PN35	Juvenile - early adolescence	Increase in PFC β-catenin levels in female rats. Decrease in PFC β-catenin in male rats. Sulpiride alleviated isolation-induced PFC β-catenin levels in females.	Funes et al., 2025
Rodent (rat)	Social isolation	PN23-PN79	Juvenile - adulthood	Decreased dendritic D2 expression in the PFC, neurons that do not receive local input from CB1-containing axon terminals.	Fitzgerald et al., 2013
Rodent (rat)	Social isolation	PN30-PN35	Juvenile - early adolescence	Decreased activity of Wnt/β-catenin pathway in the PFC	Cuesta et al., 2020
Rodent (mouse)	Social isolation	PN28-PN56	Juvenile - late adolescence	Decreased levels of dopamine receptor 2 in the medial PFC.	Zhang et al., 2021
Rodent (mouse)	Housing manipulation	PN29-PN58	Juvenile - late adolescence	Grouped and neighbor housed mice display positive discrimination index in PFC-dependent novel object recognition test. Single housed mice perform poorly in novel object recognition test.	Pais et al., 2019
Rodent (mouse)	Social isolation	PN21-PN35	Juvenile - early adolescence	Decrease in PFC-dependent social interaction and impairments to working memory, medial PFC oligodendrocytes morphological alterations, reduced myelin thickness, reduced ErbB3 signaling and reduced type III NRG1 expression.	Makinodan et al., 2012
Rodent (mouse)	Social isolation	PN31-PN56	Juvenile - late adolescence	Ventromedial PFC dendritic spine over-abundance, hyper-excitability of ventromedial PFC neurons, behavioral inflexibility and dysregulation of synaptic cytoskeletal protein ROCK2	Li et al., 2024

## 4 Discussion

### 4.1 Implications for psychiatric and neurodevelopmental disorders

Many psychiatric disorders and neurodiversities, including schizophrenia (Cheng et al., 2025; Şahin Can et al., 2025), attention-deficit/hyperactivity disorder (ADHD) (Arnsten, 2009; Jurek et al., 2025; Shaw et al., 2007), autism (Leisman et al., 2023; Patil and Kaple, 2023) and generalized anxiety disorder (Kenwood et al., 2022; McMahon et al., 2019), encompass well-documented sensory processing deficits as well as PFC dysfunction (Piccardi et al., 2021; Schwarzlose, 2026). Importantly, many of these disorders have now been described as having developmental origins, supporting the need for further research into critical periods of development. In this review, we have highlighted a myriad of studies demonstrating how distinct forms of sensory experience shape molecular, circuit, and behavioral mechanisms pivotal for PFC maturation. Collectively, this work emphasizes the need for further investigation into early interventions targeting sensory environments (Forsberg et al., 2025; Kandlur et al., 2023; Machingura et al., 2022; Piller et al., 2025; Reynolds et al., 2010; Section On Complementary Integrative Medicine et al., 2012), particularly during periods of heightened plasticity. Studies suggest that atypical sensory processing, at times alongside social and stress reactivity (Goldman-Mellor et al., 2012; Parise et al., 2025; Saavedra-Rodríguez and Feig, 2013; Zhang et al., 2019; Zorn et al., 2017), is not merely a downstream symptom of psychiatric illness but is tightly linked to symptom severity and functional outcomes, promoting the idea that sensory profiles may provide early indicators of risk and intervention targets (Bar-Shalita and Cermak, 2016; Chen Y. J. et al., 2024; Gigliotti et al., 2024; Ji et al., 2026; Piccardi et al., 2021; van den Boogert et al., 2022). However, despite growing recognition of these relationships, a major gap remains in our understanding of when these experiences exert their strongest influence on maturation of association cortices, particularly the PFC. Indeed, much of the current literature has focused on evaluating development within broadly overlapping age ranges, with limited resolution regarding which developmental stages constitute true sensitive windows versus extended periods of gradual plasticity. This challenge is further augmented by the difficulty of best aligning developmental stages across species. Given the evidence reviewed here demonstrating that sensory experience is an active determinant of PFC development, future studies should focus on a better understanding of the importance of the timing of these effects to inform preventative as well as therapeutic strategies.
